# The Effect of Electromagnetic Field Treatment on Recovery from Ischemic Stroke in a Rat Stroke Model: Clinical, Imaging, and Pathological Findings

**DOI:** 10.1155/2016/6941946

**Published:** 2016-02-01

**Authors:** Y. Segal, L. Segal, T. Blumenfeld-Katzir, E. Sasson, V. Poliansky, E. Loeb, A. Levy, A. Alter, N. Bregman

**Affiliations:** ^1^BrainQ Ltd., 9339228 Jerusalem, Israel; ^2^BioImage Ltd., 52582 Ramat Gan, Israel; ^3^Pharmaseed Ltd., 74047 Ness Ziona, Israel

## Abstract

Stroke is a leading cause of death and disability. Effects of stroke include significant deficits in sensory-motor skills and cognitive abilities. At present, there are limited effective interventions for postacute stroke patients. In this preliminary research we studied a new noninvasive, very low intensity, low frequency, electromagnetic field treatment (VLIFE), targeting a neural network, on an in vivo stroke rat model. Eighteen rats were divided into three groups: sham (M1) and two treatment groups which were exposed to VLIFE treatment for 4 weeks, one using theta waves (M2) and another using beta waves (M3); all groups were followed up for an additional month. Results indicate that the M2 and M3 treated groups showed recovery of sensorimotor functional deficits, as demonstrated by Modified Neurological Severity Score and forelimb placement tests. Brain MRI imaging results show a decrease in perilesional edema and lateral ventricle widening in the treated groups. Fiber tracts' imaging, following VLIFE treatment, showed a higher white matter integrity compared to control. Histological findings support neural regeneration processes. Our data suggest that VLIFE treatment, targeting a specific functional neural network by frequency rather than location, promotes neuronal plasticity after stroke and, as a result, improves clinical recovery. Further studies will investigate the full potential of the treatment.

## 1. Introduction

Stroke is a costly disease from human, family, and health system perspectives. Stroke is the 4th leading cause of death and a leading cause of disability in the United States [1]. Consequences of stroke include significant deficits in both sensory-motor and cognitive functioning. Stroke survivors' rehabilitation and long-term care are an ongoing significant economic burden. As a result of the ageing population, the burden of stroke will increase in the next 20 years and by the year 2030, increased cerebrovascular disease is expected to result in a tripling of stroke medical costs [2].

At present, there are limited effective interventions for patients with postacute stroke [3]. Consequently, the management of most patients with stroke remains primarily focused on secondary prevention and rehabilitation.

In the last two decades, modern noninvasive brain stimulation (NIBS) techniques have made remarkable contributions to neuroscience. Transcranial magnetic stimulation (TMS) is a noninvasive method to cause depolarization or hyperpolarization in the neurons of the brain. TMS uses electromagnetic induction to induce weak electric currents using a rapidly changing magnetic field; this can cause activity in specific or general parts of the brain with little discomfort. The method is being used both in brain function studies and as a treatment tool for various neurological and psychiatric disorders [4–6].

The two most commonly used forms of NIBS are repetitive transcranial magnetic stimulation (rTMS) and transcranial direct current stimulation (tDCS). rTMS can increase or decrease the excitability of the corticospinal tract depending on the intensity of stimulation, coil orientation, and frequency. The mechanism of these effects is not clear, though it is widely believed to reflect changes in synaptic efficacy which results in long-term potentiation (LTP) or long-term depression (LTD) [7]. Compared to TMS, tDCS is relatively safer and easier to use, and in contrast to TMS, tDCS does not result in the induction of action potentials; tDCS seems to modify the threshold for discharge of cortical neurons [8]. Despite multiple studies showing benefits of tDCS, its effectiveness in stroke rehabilitation awaits further evidence. It may have a therapeutic advantage when combined with physical rehabilitation, possibly by helping motor networks to “fine-tune” to an exercise, thereby enhancing its efficacy [9].

Results to date show that brain stimulation, as described above, has shown only modest beneficial effects on motor recovery, in the range of 10% to 30% improvement over sham treatment, in hemiplegic stroke patients [10–12].

Earlier work of Cherry [13] showed a strong scientific evidence that the human brain waves frequencies are highly correlated to the Schumann Resonance (SR) signal. The SRs are a set of spectrum peaks in the extremely low frequency (ELF) portion of the Earth's electromagnetic field spectrum. SRs are global electromagnetic resonances, excited by lightning discharges in the cavity formed by the Earth's surface and the ionosphere. SRs are the principal background in the electromagnetic spectrum beginning at 3 Hz and extend to 60 Hz, appearing as distinct peaks at extremely low frequencies (ELF) around 7.83 (fundamental), 14.3, 20.8, 27.3, and 33.8 Hz.

Electromagnetic field treatment presents a new approach to noninvasive brain stimulation.

VLIFE (Very Low Intensity and Frequency Electromagnetic Field) device, tested in this research, generates a homogeneous alternating electromagnetic field, providing whole brain stimulation. The transmission frequencies are chosen in accordance with the natural operating frequency of a desired neural network, assuming that induction of an operating frequency in a certain neural network would promote plasticity mechanisms, including neurogenesis and migration, in a specific network. The treatment aims for a functional network, unlike other stimulation methods that aim for a brain location.

We hypothesize that VLIFE treatment may enhance brain recovery after stroke and thus result in an improved clinical outcome. This is a proof-of-concept study.

## 2. Materials and Methods

### 2.1. Animals and Surgical Procedures

All experiments were carried out in accordance with the European Council Directive of November 24, 1986 (86/609/EEC) and approved by the local animal ethics committee. The animals were housed in groups (two to four animals per Type IV Makrolon cage; 60 cm long, 38 cm wide, and 20 cm high) under a 14-hour light-10-hour dark cycle with food and tap water available ad libitum. A total of 18 male Sprague-Dawley (Charles River Laboratories, Sulzheim, Germany) rats weighing 220–230 g were used. Moribund animals or animals obviously in pain or showing signs of severe and enduring distress were euthanized according to Pharmaseed SOP 005 (Euthanasia in rodent). The time of death was recorded as precisely as possible. No animal was found in a moribund or severe distress condition.

Unilateral middle cerebral artery occlusion (MCAO), a significant cause of ischemic stroke in both humans and rodents, produces contralateral neurological-sensor-motor deficits and a compensatory reliance on the less impaired side of the body ipsilateral to the injured brain. Rats were rendered hemiparesis using a tMCAO surgery, a transient middle cerebral artery occlusion.

One hour and half after occlusion rats were reanesthetized, monofilament was withdrawn to allow reperfusion, surgical wound was closed, and rats were returned to their cages (affected animals).

The most commonly used methods to monitor these sensory-motor functions include the Modified Neurological Severity Score (mNSS—a compendium of neurological neurobehavioral tests), hind limb placement, and forelimb foot fault and forelimb cylinder placement behavior [14, 15].

The day of the tMCAO surgery was defined as “Day-1,” the first treatment day was defined as “Day 1,” and termination day was defined as “Day 57.” Days 1–30 was treatment period; days 30–57 was follow-up period. The study was performed in three cycles.

Two animals died during the surgical procedure and seven after occlusion and before allocation into groups. No animal died during the VLIFE treatment period.

### 2.2. Treatment with VLIFE (Very Low Intensity and Frequency EMF)

The animals were divided into three groups. The control group received sham treatment (placed in the VLIFE device without being subjected to its alternating electromagnetic field), and two treatment groups (M2, M3). We started treatment sessions two days after tMCAO operation (day 1) and then every other day for 4 weeks. Every session lasted two minutes. Animals in group M2 were treated with alternating electromagnetic field in the frequency of theta waves (3.93 Hz) and animals in group M3 were treated with electromagnetic field in the frequency of beta waves (15.72 Hz) as described in Table 1. The animals in the sham group were placed in the VLIFE device but were not subjected to any electromagnetic field. In this experiment the lesion involved the pyramidal motor neural network; thus the treatment frequencies were chosen accordingly. Previous studies have shown that there are relatively common trends of neural activity during various movements [16, 17].

The VLIFE device generates a homogenous alternating electromagnetic field which synchronizes to a specific neural network in the brain. Therefore, the whole brain is subjected to the electromagnetic field, while only the targeted neural network is activated. The device generates a homogeneous alternating field using a coil setup known as Helmholtz coil [18].

**Figure 1:**
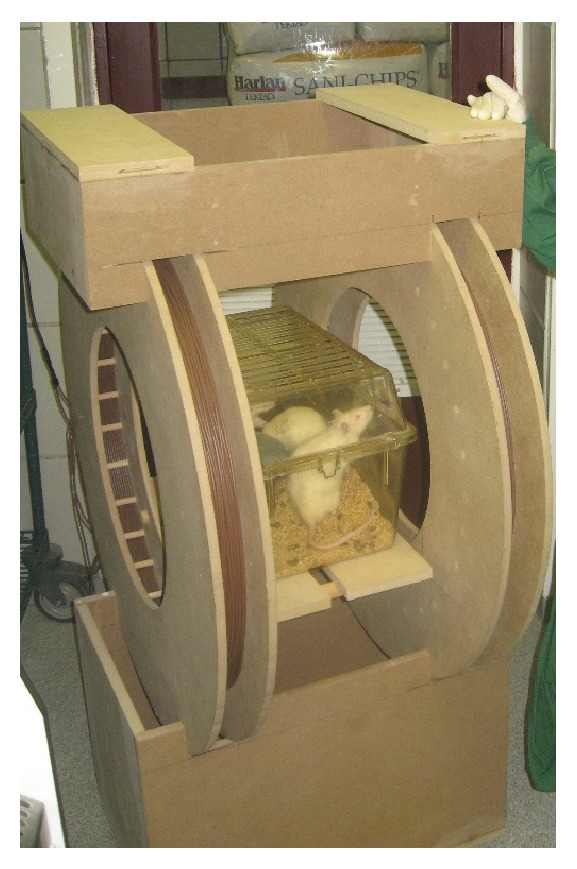


In this study VLIFE generated an electromagnetic field around the animals with intensity ranges within ±0.5 Gauss, similar intensity to the geomagnetic field [19] (see Figure 1).

### 2.3. Body Weight and Neurological Scores

#### 2.3.1. Body Weight

Body weight was monitored before tMCAO and at days 11, 19, 27, 35, 43, and 57.

#### 2.3.2. Modified Neurological Severity Score (mNSS)

The mNSS is a composite of the motor (muscle status, abnormal movement), sensory (visual, tactile, and proprioceptive), and reflex tests. Modified NSS evaluation was performed at days 1, 11, 19, and 27 which are in the treatment period and days 35, 43, and 57 which are in the follow-up period, as described previously in order to grade poststroke sensory-motor neurologic deficits [20].

Animals with an overall score of less than 10 were excluded from the study. Paired, two-tailed *t*-test was performed comparing treatment group to sham group.

#### 2.3.3. Forelimb Placement Test (FPT)

FPT, a test detecting sensory-motor deficit as well as severe loss of interhemispheric sensory-motor integration, was measured/scored at days 1, 11, 19, 27, 35, 43, and 57 afterwards. Each rat was placed in an upright Plexiglas cylinder open at both ends and measuring 30 cm high by 20 cm in diameter placed open end down on a table (i.e., confining the rat being tested within). The number of each forelimb, or both forelimb placements on the wall of the cylinder, was recorded [21]. Paired, two-tailed *t*-test was performed comparing treatment group to sham group.

### 2.4. Brain Sectioning and Staining

#### 2.4.1. BrdU Staining

Bromo-Deoxyuridine (BrdU) administration was performed according to Pharmaseed SOP #037 (BrdU Handling). BrdU was administered intraperitoneally (IP) twice daily in an interval of about 8 hours on days 4–8 and day 32. Dose volume was 1 mL/kg.

Animals for MRI measurements were not administered BrdU.

On day 57 animals were anesthetized by Pental (60 mg/kg) and perfusion was performed as follows: 500 mL/kg of cold heparinized saline (10 Unit/mL), followed by 250 mL/kg of cold 4% Paraformaldehyde (PFA).

#### 2.4.2. Tissue Harvesting and Processing for Histology and IF Immunohistochemistry (IHC)

Brains were collected from 9 animals and were immersed in 4% freshly prepared PFA for 24 hours. Then the brain tissues were transferred into a 1.25% PFA solution.

#### 2.4.3. Brains for IHC

The rat brains were cryoprotected by submerging them in sequence of 10% sucrose solution for 1 hour; 20% sucrose solution for 1 hour; and 30% sucrose for 1 hour or until the brains completely sink (times may be longer for larger brains). Finally, the brains were placed into 50/50 (30% sucrose/O.C.T. embedding medium) for 15 minutes. The rat brains were cut systematically in transversal cross sections. The sections were kept in 24-well plates and divided into coordinates for later orientation.

All slides were stained with BrdU IHC combined with one of the markers GFAP, Nestin, or Double-Cortin, using free floating sections and were examined by one pathologist. Proliferation scoring grades were held from 0 to 4:

 0 = proliferation grade that is comparable to the negative control group.
 1 = very mild proliferation with up to 1–25% increase in cell number per HPF.
 2 = mild proliferation with up to 26–50% increase in cell number per HPF.
 3 = moderate proliferation with up to 51–75% increase in cell number per HPF.
 4 = strong proliferation with up to 76–100% increase in cell number per HPF.

Next to BrdU, other markers including GFAP, Nestin, and Double-Cortin were evaluated as double staining. Scoring was as follows:

 0 = no positive reaction at all.
 1 = very mild reaction (1–5 positive cells per 20x HPF).
 2 = mild reaction (5–10 positive cells per 20x HPF).
 3 = moderate reaction (10–20 positive cells per 20x HPF).
 4 = strong reaction (20–50 positive cells per 20x HPF).

### 2.5. Imaging: MRI Protocols and Analysis

The rats were scanned in the MRI in four time points: 1 day after tMCAO which is one day before VLIFE treatment began (TP1), two weeks after the first treatment (TP2), 1 month after the first treatment (TP3), and 2 months after the first treatment which is one month after the final treatment session (TP4).

Three rats were scanned in a 7 T MRI system, Bruker, Germany using a quadrate head coil. T2 weighted imaging was performed in a T2 parameters: MSME sequence, TR = 3500 ms, 16 different TE (ms): 10, 20, 30, 40, 50, 60, 70, 80, 90, 100, 110, 120, 130, 140, 150, and 160, and spatial resolution: 0.07 × 0.07 × 0.8 mm. Diffusion tensor imaging (DTI) was performed with the following parameters: TR/TE = 7500/25 ms, 4 EPI segments, Δ/*δ* = 10/4.5 ms, 15 noncollinear gradient directions with a single *b* value shell at 1000 sec/mm^2^ and one image with *b* value of 0 sec/mm^2^ (referred to as *b*_0_), 2-3 repetitions. Geometrical parameters were 30 slices of 0.8 mm thickness (brain volume) and in-plane resolution of 0.156 × 0.156 mm^2^ (matrix size of 128 × 128 and FOV of 16 mm^2^).

Fiber tracking was performed using Explore DTI software [22]. The tensors obtained were spectrally decomposed to their eigen-components. The eigen-values were used to calculate FA maps [23]. Tractography was applied using Deterministic (streamline) fiber tracking, terminating at voxels with FA lower than 0.15 or following tract orientation change higher than 20°. Fibers that passed through a manually chosen seed region of interest (ROI) were plotted. The fibers were plotted as streamlines. The masks obtained were overlaid over the color-coded FA image. Overall four fibers tracts were plotted for each rat in each time point: corpus callosum, internal capsule (left and right), fornix-fimbria, and anterior commissure (left and right). The average FA and MD values were extracted in each fiber tract. Reconstruction of four fiber tracking systems of the brain was performed, using advanced image postprocessing analysis: the corpus callosum, internal capsule, fornix-fimbria, and the anterior commissure (not shown).

For image analysis, T2 relaxation maps, ADC and FA maps were calculated in MATLAB (©Mathworks, USA) and using BioImage software. For ROI analysis, edema and regions of interest (ROI) were outlined manually in MATLAB (©Mathworks, USA) using BioImage software. Edema was outlined manually in regions showing high T2 intensity, and an equivalent region was marked in the left hemisphere. The average T2 and DTI values were extracted in all the outlined regions. The brain ventricles, edema, and contrahemisphere volumes were measured in each time point outline performed on the T2 maps using BioImage software.

## 3. Results

### 3.1. Body Weight

Body weight increased in all groups with no statistically significant differences between the groups.

### 3.2. Clinical Tests

**Figure 2:**
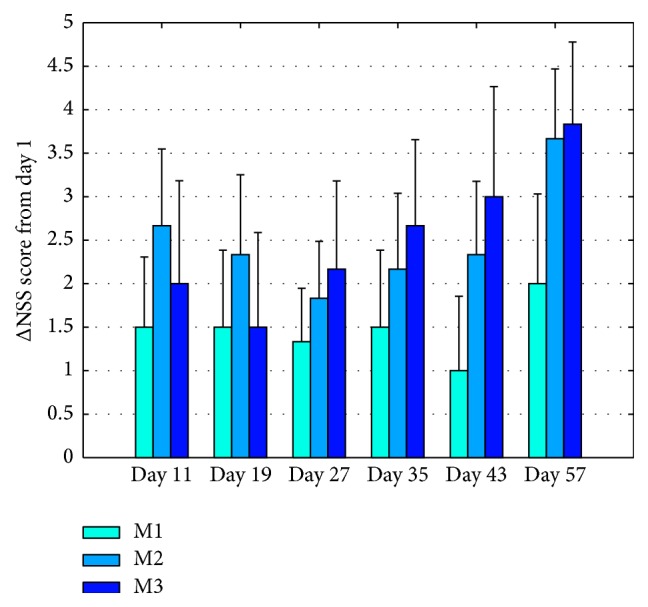
Modified Neurological Severity Scale. Modified Neurological Severity Score was tested in 15 animals at days 1, 11, 19, 27, 35, 43, and 57. M1 group received sham treatment, M2 was exposed to 3.93 Hz, and M3 was exposed to 15.72 Hz VLIFE 2 min sessions, in alternate days, for a month and followed up for an additional month. Values are mean ± SE of the difference from day 1; *p* = 0.002 for M2 and *p* = 0.02 for M3.

Modified NSS scores, reflecting motor deficit recovery, are presented in Figure 2. M2 and M3 treatment groups showed a significant increased recovery in sensory-motor functional deficits (*p* = 0.002, *p* = 0.02, resp.).

**Figure 3:**
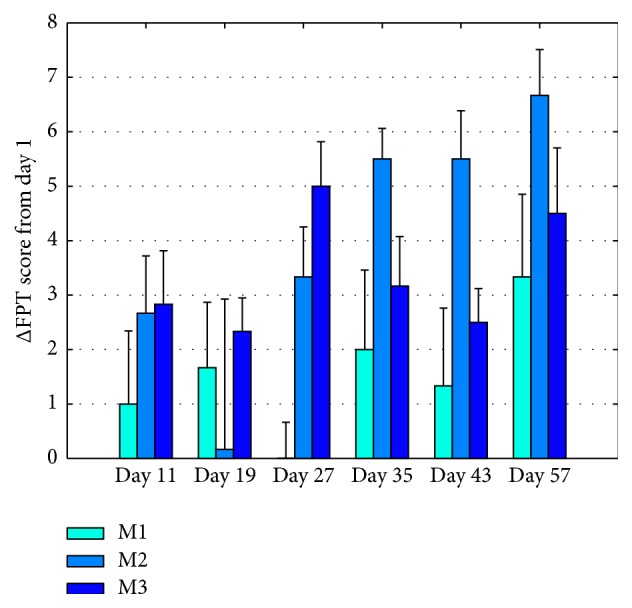
Forelimb placement test. Forelimb placement, reflecting sensorimotor deficit recovery, was tested in 15 animals at days 1, 11, 19, 27, 35, 43, and 57. M1 is a group receiving sham treatment, M2 was exposed to 3.93 Hz and M3 was exposed to 15.72 Hz VLIFE 2 min sessions, in alternate days, for a month and followed up for an additional month. Values are Mean ± SE of FPT difference from day 1. *p* = 0.036 for M2 and *p* = 0.04 for M3.

Results of the FPT, presented in Figure 3, demonstrate a significant recovery of sensory-motor deficit in the M2 and M3 treated rats (*p* = 0.036, *p* = 0.04, resp.); these results support, together with Figure 2 results, a significant clinical improvement.

### 3.3. MRI Images and Analysis

#### 3.3.1. Volumetric Analysis

**Figure 4:**
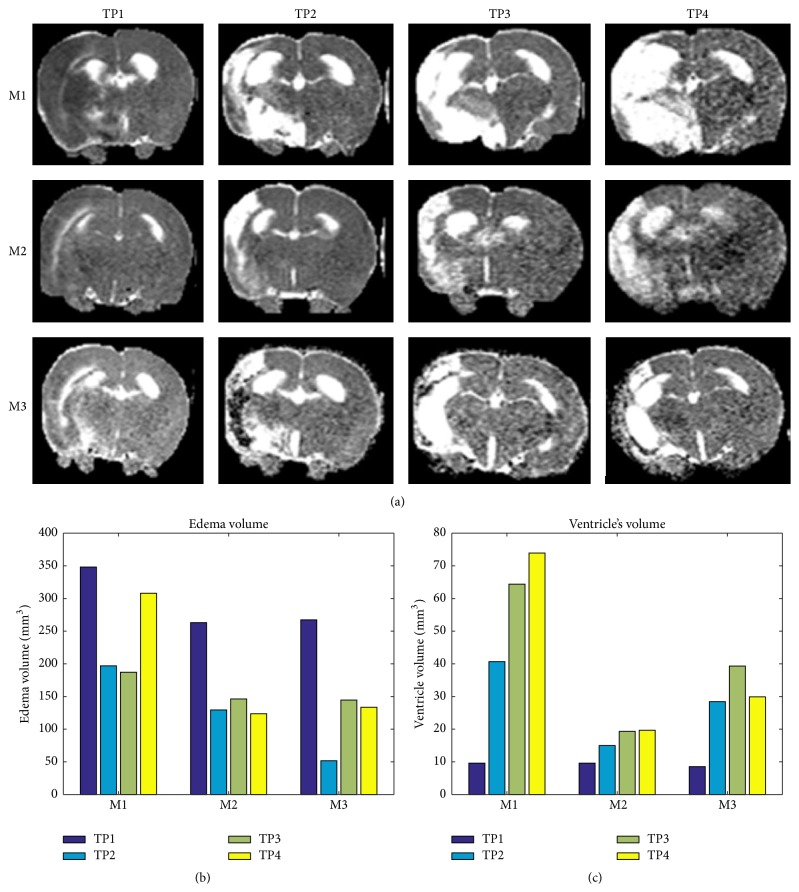
Edema and right ventricle's volume (affected hemisphere). The edema region was measured in each time point in the T2 maps, the region of high intensity T2 was measured in all slices (a). Quantitative data of edema's volume (b) and right ventricle's volume (c) is shown in each rat at each time point in one representative MRI slice. One day after tMCAO (TP1), two weeks after the first treatment (TP2), 1 month after the first treatment (TP3), one month after the final treatment session (TP4). M1 is a group receiving sham treatment, M2 was exposed to 3.93 Hz, and M3 was exposed to 15.72 Hz VLIFE 2 min sessions, in alternate days, for a month and followed up for an additional month.

Edema and ventricles volumetric analyses were performed as described in Figure 4.

Results show that the edema and right ventricle volume decreased substantially in the treated rats (M2, M3) compared with sham (M1).

**Figure 5:**
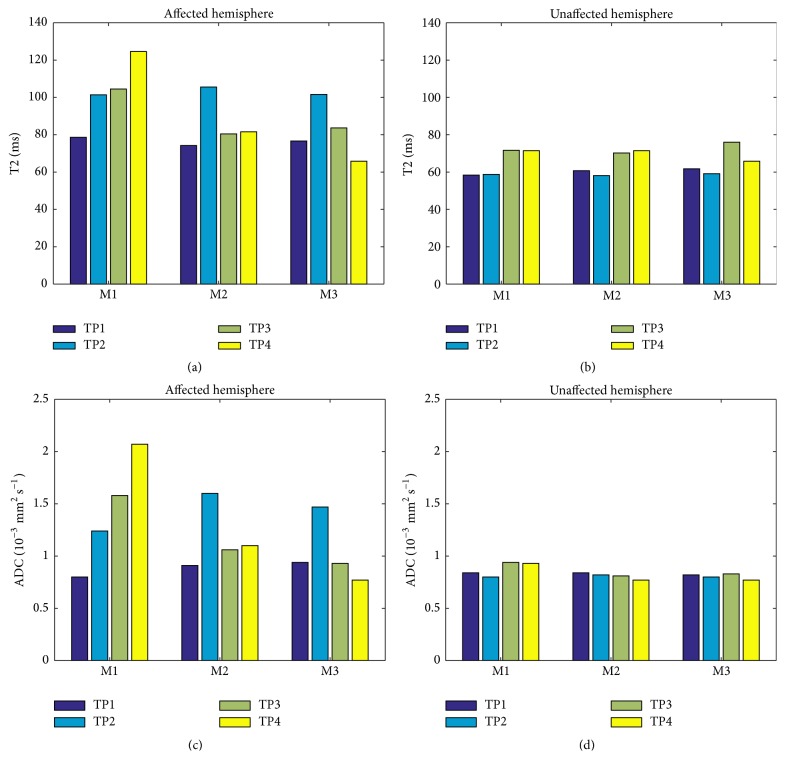
Measured T2 and ADC of the affected and unaffected hemispheres. A quantitative evaluation from MRI T2 sequence ((a), (b)) and ADC (Apparent Diffusion Coefficient) sequence ((c), (d)) maps of each rat during the different time points. The right (affected) and left (unaffected) hemispheres were compared at different time points as described in Figure 4 (TP1–TP4) in untreated animal group (M1) and treated groups as described in Figure 4 (M2, M3).

Figure 5 presents a quantitative evaluation from MRI T2 sequence and ADC sequence during the different time points. Affected and unaffected hemispheres were compared, at time points TP1–TP4 as described in Section 2.5. One can observe in the unaffected hemisphere no significant value changes in both parameters at all time points and among all the animals, while in the affected hemisphere, T2 and ADC measurements were higher in the untreated animal in the last two time points.

**Figure 6:**
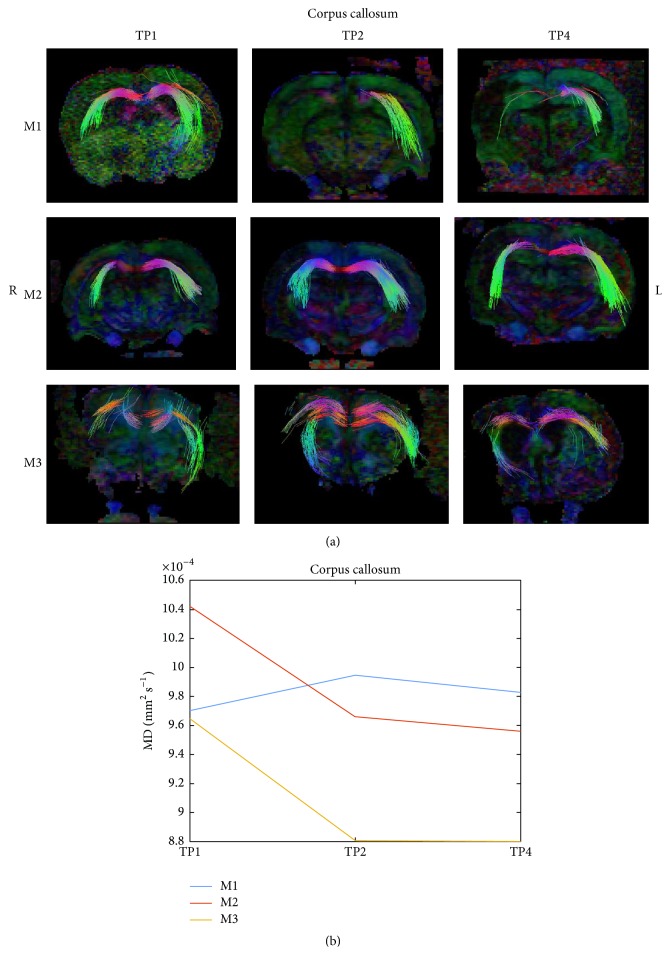
DTI of corpus callosum. A reconstruction of the corpus callosum white matter system (a) of the 3 examined rat groups (M1, M2, and M3 as described in Figure 4) at 3 time points (TP1, TP2, and TP4 as described in Figure 4). The median diffusivity of the fiber system was extracted and is shown in (b).

Figure 6 presents a reconstruction of the corpus callosum white matter system of the 3 examined rat groups at 3 time points. Fiber tracking visualized fewer fibers in the untreated rat compared to the treated rats. Diffusivity parameter of the corpus callosum increased in the untreated rat M1 and decreased in the treated rats M2, M3.

**Figure 7:**
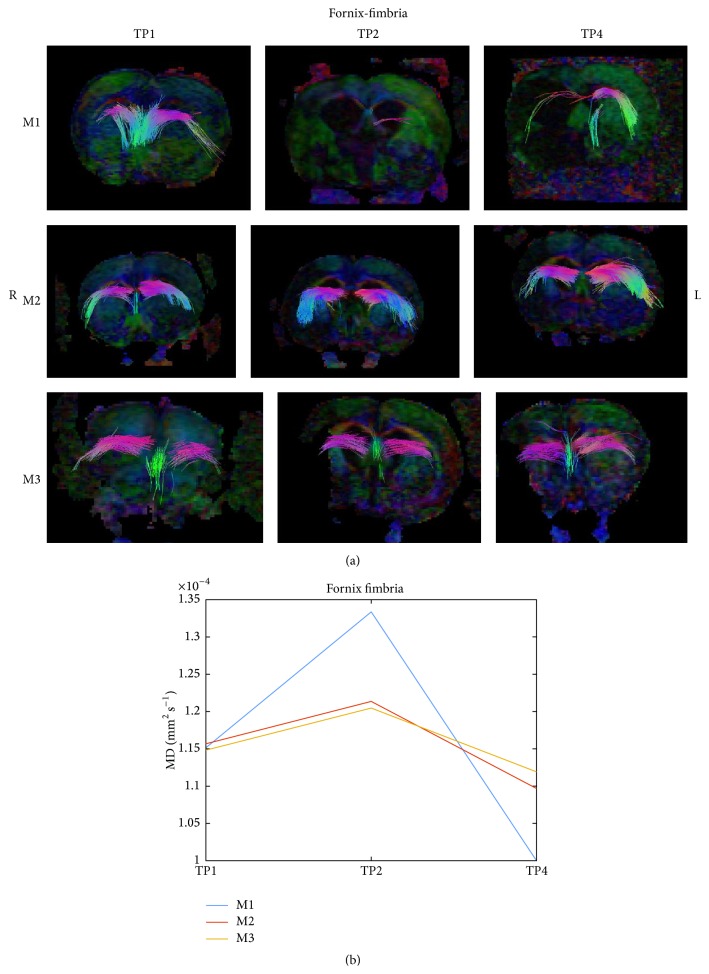
DTI of fornix-fimbria. A reconstruction of the fornix-fimbria white matter system (a) of the 3 examined rat groups (M1, M2, and M3 as described in Figure 4) at 3 time points (TP1, TP2, and TP4 as described in Figure 4). The median diffusivity of the fiber system was extracted and is shown in (b).

Figure 7 presents a reconstruction of the fornix-fimbria white matter system (a) of the 3 examined rat groups (M1, M2, and M3 as described in Table 1) at 3 time points (TP1, TP2, and TP4 as described in Section 2.5). Fiber tracking visualized fewer fibers in the untreated rat compared to the treated rats. Diffusivity parameter of the fornix-fimbria decreased in all rats, but to less extant in the untreated rat.

**Figure 8:**
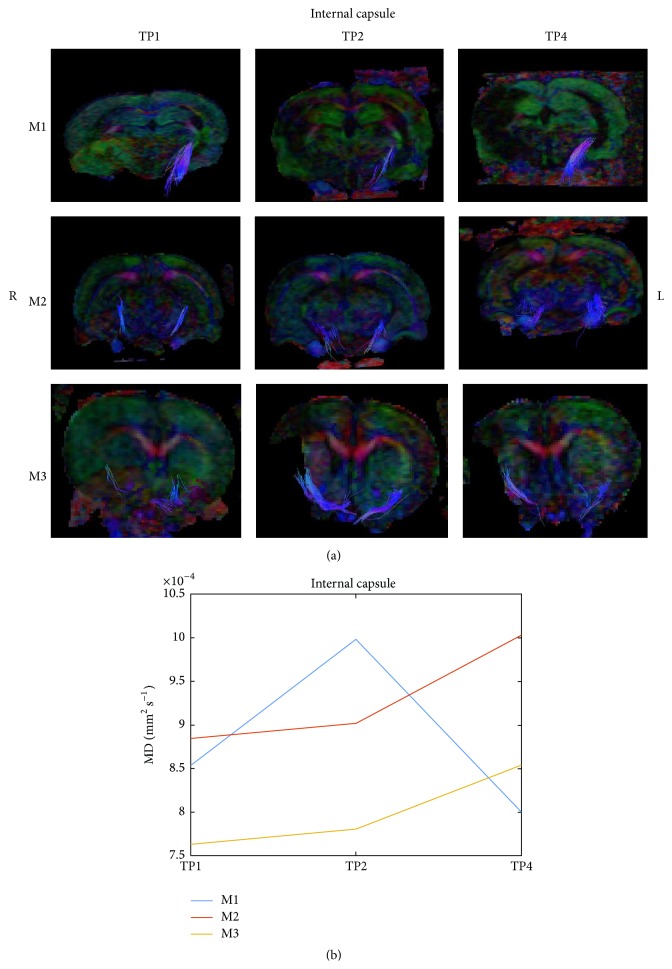
DTI of internal capsule. A reconstruction of the internal capsule white matter system (a) of the 3 examined rat groups (M1, M2, and M3 as described in Figure 4) at 3 time points (TP1, TP2, and TP4 as described in Figure 4). The median diffusivity of the fiber system was extracted and is shown in (b).

Figure 8 presents a reconstruction of the internal capsule white matter system (a) of the 3 examined rat groups (M1, M2, and M3 as described in Table 1) at 3 time points (TP1, TP2, and TP4 as described in Section 2.5). Fiber tracking visualized fewer fibers in the untreated rat compared to the treated rats. Diffusivity parameter of the fornix-fimbria decreased in the control rat and increased in the treated rats.

### 3.4. Histopathological Findings

Table 2 summarizes all histopathological findings. Histopathology procedures were performed on 3 random animals out of 5 at each group.

BrdU was strongly expressed in all animals in the infracted and in the periventricular areas. GFAP was expressed only at the infarct area in all animals. A rough estimation showed that 40–70% of the BrdU positive cells turned out to be GFAP positive cells. Few cells positive for Nestin were found in two and three animals from treatment groups M2 and M3, respectively, in the infarcted and periventricular areas. Also, few cells were found to be positive for DC in the periventricular area, in one animal from group M2 and one from group M3. Results show that progenitor cells migrated to the margins of the affected areas in the treated groups.

## 4. Discussion

In this proof-of-concept study, we investigated the effects of multisession low intensity and frequency electromagnetic field in a juvenile rat stroke model in vivo, on clinical improvement and brain response to stimulation, using MRI imaging and histological dyeing techniques.

The present study indicates a significant clinical improvement in the groups treated with 3.93 Hz and 15.72 Hz as demonstrated by NSS and forelimb placement test results that reflect recovery of sensory-motor functional deficits.

Brain MR imaging shows encouraging results in both treated groups. Decreased perilesional edema volume and lateral ventricle widening (as a marker for secondary atrophy) were detected. Fiber tracts imaging following VLIFE treatment showed higher white matter integrity compared to control as demonstrated in Figures 6, 7, and 8. Histological findings support neural regeneration processes, showing positive cells for Nestin and Double-Cortin (DCX) in the treated groups, both neural progenitor cell markers as shown in Table 2.

The improvement of white matter integrity with time could be due to axonal growth and increased myelin integrity with generation of new myelin sheaths. The results could also be explained by reduction in the edema which was quantified previously based on the T2 images. These hypotheses could be further supported using histology staining for myelin basic protein.

Histological findings, combined with the clinical improvement suggest that even though the new brain cells and the newly white matter connecting them are located in similar regions of the brain, the activities which were improved are different, supporting the hypothesis that each nervous system operates on a different frequency, and were enhanced by a different treatment frequency accordingly. This may lead in the future to personal dedicated treatment protocols as part of rehabilitation procedures after stroke.

Preliminary findings are presented here, and further studies may allow better understanding regarding brain recovery mechanisms enhanced by poststroke VLIFE electromagnetic field treatment and its potential.

Our data may suggest that VLIFE treatment promotes neuronal plasticity after stroke and, as a result, promotes sensory-motor recovery.

VLIFE and VLIFE treatment are patent-pending.

## Figures and Tables

**Table 1:** Groups and treatment allocation.

Group number	*n*	tMCAO	Electromagnetic field treatment_(intensity±0.5 Gauss)_	
			Duration	Frequency
M1 control	*n* = 6	Day-1	NA	NA
M2 treatment	*n* = 6		2 min	3.93 Hz
M3 treatment	*n* = 6			15.72 Hz

Treatment consisted of 2 min session delivered in alternate days for a month to the whole body. M1 animals #: 1, 2, 26, 29, 34, and 39. M2 animals #: 4, 7, 20, 24, 40, and 47. M3 animals #: 11, 22, 23, 25, 38, and 45.

Animals 39, 47, and 45 were scanned with MRI.

**Table 2:** Histopathological findings.

Animal	Group	Location of positive BrdU	Location of positive Nestin	Location of positive DC	Location of positive GFAP
#2	M1	Infarct + ventricle	—	—	Infarct
#26	M1	Infarct + ventricle	—	—	Infarct
#29	M1	Infarct + ventricle	—	—	Infarct
#20	M2	Infarct + ventricle	Ventricle	Ventricle	Infarct
#40	M2	Infarct + ventricle	—	—	Infarct
#7	M2	Infarct + ventricle	Ventricle	—	Infarct
#11	M3	Infarct + ventricle	Ventricle	—	Infarct
#38	M3	Infarct + ventricle	Infarct	Ventricle	Infarct
#25	M3	Infarct + ventricle	Infarct	—	Infarct

M1 group received sham, M2 received 3.93 Hz treatment, and M3 received 15.72 Hz treatment. BrdU, detecting cell proliferation. GFAP, detecting CNS cells. Nestin, detecting brain stem cells. Double-Cortin, detecting progenitor cells.
